# Cellular stress increases DRIP production and MHC Class I antigen presentation

**DOI:** 10.3389/fimmu.2024.1445338

**Published:** 2024-08-23

**Authors:** Natalie Pach, Michael Basler

**Affiliations:** ^1^ Institute of Cell Biology and Immunology Thurgau (BITG) at the University of Konstanz, Kreuzlingen, Switzerland; ^2^ Division of Immunology, Department of Biology, University of Konstanz, Konstanz, Germany

**Keywords:** defective ribosomal product, DRIP, antigen presentation, antigen processing, LCMV, ISG15, protein degradation, MHC

## Abstract

**Background:**

Defective ribosomal products (DRiPs) are non-functional proteins rapidly degraded during or after translation being an essential source for MHC class I ligands. DRiPs are characterized to derive from a substantial subset of nascent gene products that degrade more rapidly than their corresponding native retiree pool. So far, mass spectrometry analysis revealed that a large number of HLA class I peptides derive from DRiPs. However, a specific viral DRiP on protein level was not described. In this study, we aimed to characterize and identify DRiPs derived from a viral protein.

**Methods:**

Using the nucleoprotein (NP) of the lymphocytic choriomeningitis virus (LCMV) which is conjugated N–terminally to ubiquitin, or the ubiquitin-like modifiers FAT10 or ISG15 the occurrence of DRiPs was studied. The formation and degradation of DRiPs was monitored by western blot with the help of a FLAG tag. Flow cytometry and cytotoxic T cells were used to study antigen presentation.

**Results:**

We identified several short lived DRiPs derived from LCMV-NP. Of note, these DRiPs could only be observed when the LCMV–NP was modified with ubiquitin or ubiquitin-like modifiers, but not in the wild type form. Using proteasome inhibitors, we could show that degradation of LCMV-NP derived DRiPs were proteasome dependent. Interestingly, the synthesis of DRiPs could be enhanced when cells were stressed with the help of FCS starvation. An enhanced NP118–126 presentation was observed when the LCMV-NP was modified with ubiquitin or ubiquitin-like modifiers, or under FCS starvation.

**Conclusion:**

Taken together, we visualize for the first time DRiPs derived from a viral protein. Furthermore, DRiPs formation, and therefore MHC-I presentation, is enhanced under cellular stress conditions. Our investigations on DRiPs in MHC class I antigen presentation open up new approaches for the development of vaccination strategies.

## Introduction

The reliable transfer of genetic information from DNA to RNA and finally to protein is crucial for cellular life. Nevertheless, errors can occur during transcription and translation resulting in the production of unfunctional or truncated proteins, which are unable to achieve their native structure, thus affecting protein homeostasis (1). Impaired protein homeostasis then can lead to cell dysfunction, aging, and disease (2). First conceived by Yewdell et al. in 1996, defective ribosomal products (DRiPs) were introduced as a source of endogenous antigens for presentation on MHC class I molecules (3). DRiPs emerge from premature termination during translation, defective mRNAs, frame shifting, tRNA-amino acid misacylation and all other errors that occur during translation (4–6). Two potential sources contribute to MHC class I antigenic peptides: 1. retirees and 2. DRiPs (7, 8). Retirees are degraded proteins that reach metabolic stability, whereas DRiPs degrade more rapidly than their corresponding native retiree pools. DRiPs belong to the group of rapidly degraded proteins (9). 30% of all newly synthesized proteins in mammalian cells are degraded within 30 minutes by the proteasome and, therefore, are assumed to arise from DRiPs. It was found that most antigens presented on MHC class I molecules do not derive from the protein turnover of retirees, but from DRiPs (10, 11). Khan et al. could demonstrate that the direct presentation of antigens derived from long–lived viral proteins on MHC class I strongly relies on neosynthesis (12, 13). Due to coupling of neosynthesis and degradation, DRiPs facilitate a rapid response of CD8+ T cells to infected cells (14). Indeed, it has recently been shown that ribosome-associated quality control is involved in generating epitopes for antigen presentation on MHC class I molecules (15). N-terminal fusion of the ubiquitin-like modifier (ULM) ISG15 to the nucleoprotein of the lymphocytic choriomeningitis virus (LCMV) leads to the degradation of the fusion protein in a proteasome-independent manner (16). However, in the same study, using the proteasome inhibitor bortezomib antigen presentation of LCMV-derived peptides from the ISG15-NP fusion protein were proteasome dependent and enhanced compared to the non-fused NP, supporting that DRiPs are the major source of this viral fusion protein (16). In a different study, N-terminal fusion of the ubiquitin-like protein neural precursor cell expressed developmentally down-regulated protein 8 (NEDD8) initiates the destruction of the fused protein (17). In addition, inhibition of NEDDylation only affected MHC-I presentation of DRIPs but not retirees, emphasizing the importance of DRiPs in pathways of antigen presentation (18).

Cell stress affects the cellular equilibrium and thus, the translation fidelity, leading to enhanced DRiP formation (19). During cellular stress, DRiPs could accumulate in the ER and are recognized by specific proteins of the quality control machinery. Important players are chaperones belonging to the HSP70 family, which can bind co-translationally to nascent polypeptides, and valosin-containing protein (VCP) (20). After binding, both proteins, HSP70 and VCP, could assist with the ubiquitination of DRiPs, facilitating their degradation by the proteasome to restore cellular homeostasis (9, 21).

Interestingly, although DRiPs represent a major source of peptides used for the presentation by MHC class I, the molecular mechanisms leading to processing and presentation remain largely unclear.

In this study, we investigated the influence of different cellular stress conditions on DRiP formation and DRiP-derived antigen presentation. We found that DRiP formation is induced by N–terminal modifications of the stable viral nucleoprotein of LCMV and can be monitored on protein level. Furthermore, we report that elevated levels of DRiPs are achieved by cellular stress, increasing MHC-I antigen presentation of LCMV-derived epitopes. Taken together, these data strongly support the concept of DRiPs as an essential MHC class I ligand pool.

## Results

### N-terminal conjugation of ubiquitin and ULMs leads to production of fragments degraded by the proteasome

**Figure 1:**
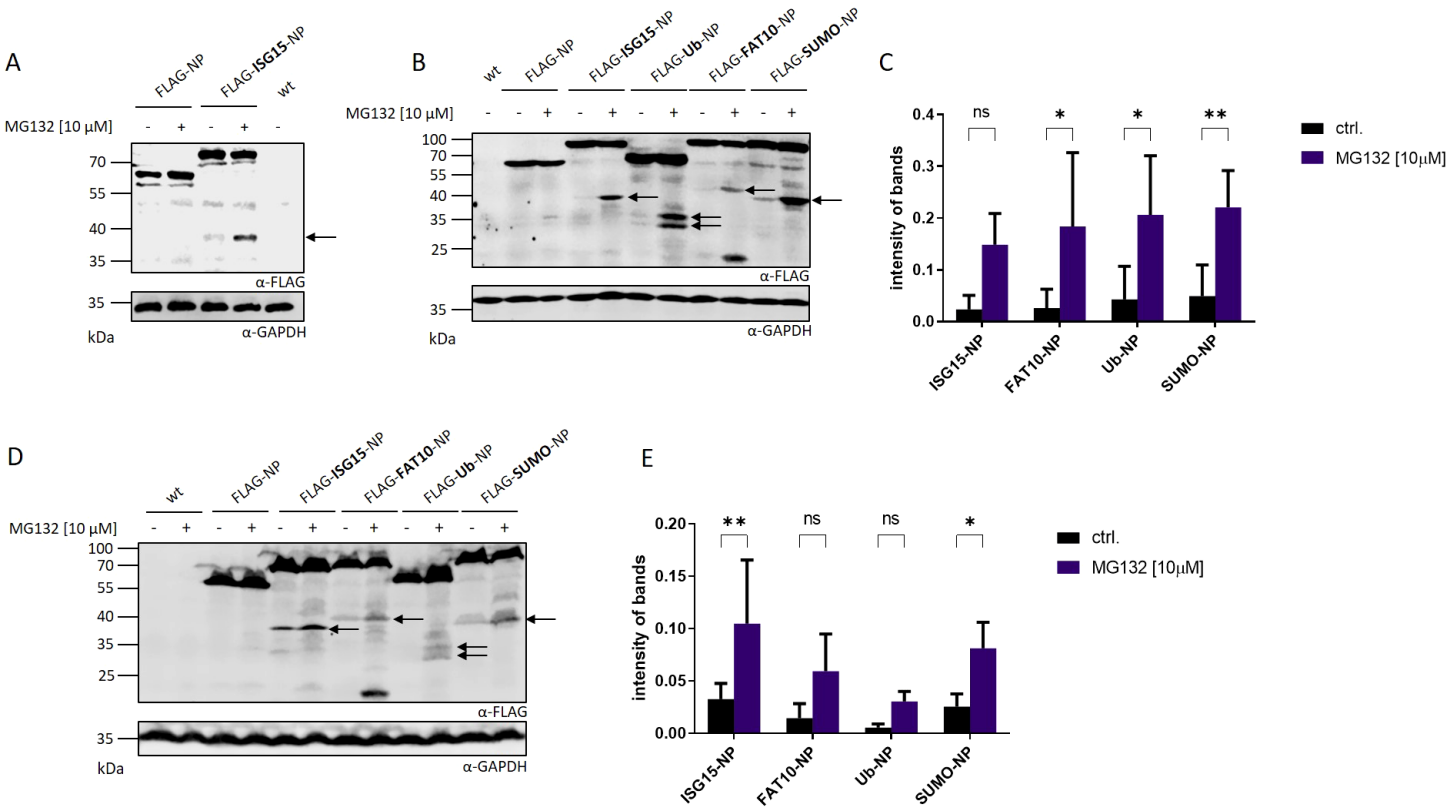
Detection of proteasome-dependent fragments after N-terminal modification of the LCMV nucleoprotein. **(A)** HEK293T cells were transiently transfected with expression constructs for FLAG-tagged LCMV nucleoprotein (FLAG-NP) or FLAG-tagged ISGylated nucleoprotein (FLAG-ISG15-NP) and analyzed by western blot using an anti-FLAG antibody (upper western blot). **(B)** Representative FLAG western blot showing small fragments after transient transfection of HEK293T cells with indicated expression constructs. **(C)** Graph shows the quantification of the FLAG signal detected for the observed fragments normalized to GAPDH as the mean ± SD derived from three independent experiments (n=3). **(D)** HeLa cells were transiently transfected with indicated expression constructs and analyzed by western blot using an anti-FLAG antibody (upper western blot). **(E)** Graph shows the quantification of FLAG signals detected for the observed fragments normalized to GAPDH as the mean ± SD derived from three independent experiments (n=3). **(A, B, D)** Where indicated (+) proteasome activity was inhibited by adding MG132 at a concentration of 10 µM for the last 5 hours. Untransfected cells (indicated wt) were used as negative control. Arrows indicate DRiP fragments. GAPDH was used as loading control (lower western blot). Representative western blots of three independent experiments are shown (n = 3). **(C, E)** Two-way ANOVA with Sidak**’**s multiple comparisons test, *p < 0.05; **p < 0.01; ns: not significant was applied for statistical analysis.

The involvement of ISG15 in the formation of DRiPs remained unclear. Held et al. could show that the degradation of the full-length ISG15-LCMV-NP fusion protein (ISG15-NP) was proteasome independent (16). In contrast, the presentation of ISG15-NP derived peptides on MHC-I was strongly dependent on the proteasome, indicating that ISG15-NP derived MHC-I peptides do not derive from the fully-folded protein but emerge from DRiPs (16). Therefore, the degradation and DRiP formation of ISG15-NP was further investigated in this study. To analyze degradation products of ISG15-NP an N-terminally FLAG tagged ISG15-NP (FLAG-ISG15-NP) was generated and compared with FLAG-NP. HEK293T cells were transiently transfected with plasmids encoding FLAG-ISG15-NP or FLAG-NP and analyzed by western blot. To visualize short-lived fragments resulting from aborted translation and thereby representing DRiPs, proteasome inhibition (MG132) was used. (**Figure 1A**). Transfection of both, FLAG-NP and ISG15-FLAG-NP, resulted in the expression of the full-length protein. Interestingly, in contrast to the unmodified LCMV nucleoprotein, expression of FLAG-ISG15-NP showed the formation of a small fragment, which was only visible when proteasome activity was inhibited. To further investigate whether the occurrence of the proteasome-dependent fragment observed in **Figure 1A** was dependent on ISG15 or can be induced by other modifiers, ubiquitin (FLAG-Ubi-NP), human leukocyte antigen (HLA)-F adjacent transcript 10 (FAT10) (FLAG-FAT10-NP) and small ubiquitin-like modifier (SUMO) (FLAG-SUMO-NP), were fused N-terminally to the LCMV nucleoprotein. Except for the nucleoprotein alone (FLAG-NP), all fusion proteins showed, apart from the full-length protein, an additional fragment of low molecular weight in the western blot, which could be stabilized with MG132 (**Figures 1B, C**). Since the fragments strongly accumulate when proteasome activity is inhibited for 5 hours, this indicates that the observed fragments, in contrast to the full length-proteins, are short-lived and degraded by the proteasome. The peptide-aldehyde proteasome inhibitor MG132 has off-target effects (22, 23) and can affect many cellular processes such as cell cycle and apoptosis (24, 25). To exclude off-target effects of MG132 on non-proteasomal targets, cells were further treated with lactacystin, an irreversible proteasome inhibitor binding to the beta subunits of the 20S proteasome. Western blot analysis showed that fragments could be stabilized with both inhibitors in the same manner validating that the degradation of the small fragments is proteasome dependent (**Supplementary Figure 1**). Interestingly, additional, less prominent fragments, stabilized by proteasome inhibition, could be observed in this western blot. In addition, a prominent small fragment at approximately 20 kDa could be observed for FLAG-FAT10-NP, which fits the molecular weight of FAT10 (**Figure 1B**). Additionally, for FLAG-Ub-NP, two less pronounced small fragments could be observed, both accumulating in the presence of MG132. So far, HEK293T cells were used for transfection of plasmids encoding the different NP constructs. To check whether the production of the smaller fragments relies on a specific cell type, transfection was performed using HeLa cells. Expression of proteins was analyzed via western blot and inhibition of proteasomal activity was achieved with MG132 (**Figures 1D, E**). Using a different cell type led to the production of the same fragments seen in HEK293T cells (**Figures 1B, D**). All of the small fragments occurring after N-terminal modification of the NP are of the same molecular weight as compared to the fragments observed in HEK293T cells (**Figures 1B, D**). Furthermore a stabilization of the fragments could be achieved upon proteasome inhibition using MG132 (**Figures 1B, D**). These data show that the production of the observed fragments is cell type independent. Hence, the production of the observed NP fragments are not cell type specific. In summary, N-terminal conjugation of ubiquitin and ULMs to the stable viral nucleoprotein of LCMV leads to the production of small fragments degraded by the proteasome.

### Smaller fragments arise due to termination of translation

**Figure 2:**
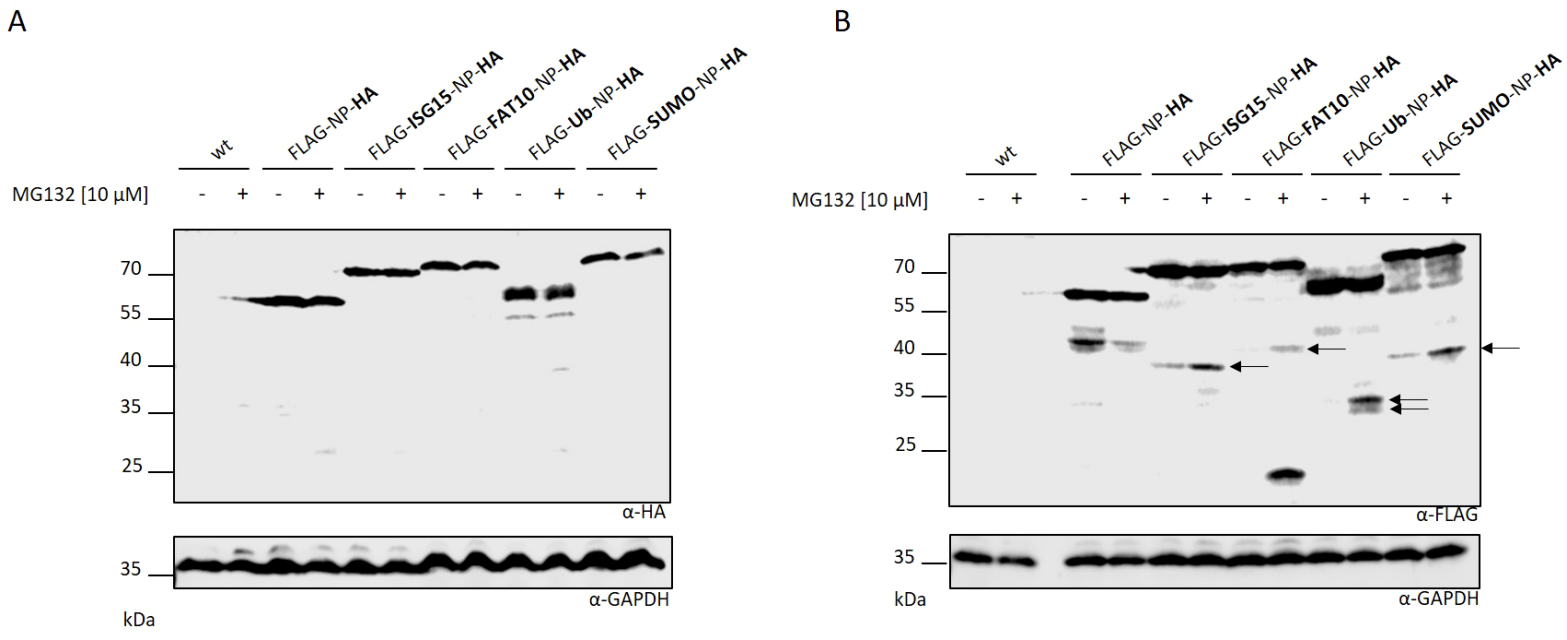
Fragments are produced as a result of aborted translation. HEK293T cells were transiently transfected with indicated expression constructs carrying an additionally HA tag at the C-terminus. Western blot was performed using an **(A)** HA or **(B)** FLAG specific antibody. **(A, B)** Where indicated (+) proteasome activity was inhibited by adding MG132 at a concentration of 10 µM for the last 5 hours. Untransfected cells (indicated wt) were used as negative control. GAPDH was used as loading control (lower western blot). Arrows indicate DRiP fragments.

Next, we aimed to investigate whether the observed protein fragments resulted from abortion of protein translation or enzymatic cleavage post-synthesis. Therefore, we cloned plasmids encoding an HA tag at the C-terminus of the different LCMV nucleoprotein constructs. HEK293T cells were transiently transfected with the plasmids and the protein expression was monitored via immunoblotting (**Figure 2A**). No C-terminal fragments could be observed in western blot analysis using an HA-specific antibody. This data indicates that the N–terminal fragments observed in **Figure 1** do not derive from the full length protein. In case of enzymatic cleavage post-synthesis, the detection of a N- and C-terminal fragment would have been expected. Hence, this indicates a premature termination of the modified LCMV-NP during protein translation. To ensure that the additional HA tag at the C-terminus had no influence on the fragment formation observed in **Figure 1**, an additional immunoblotting against the FLAG tag was performed (**Figure 2B**). A similar fragment pattern as seen in **Figure 1** was observed, excluding an influence of the HA tag on the DRiP fragment formation. Taken together, the fragments, which are degraded by the proteasome, are a consequence of a premature translation termination rather than an enzymatic cleavage subsequent to synthesis.

### Fragments are short-lived and degraded in a proteasome-dependent manner

**Figure 3:**
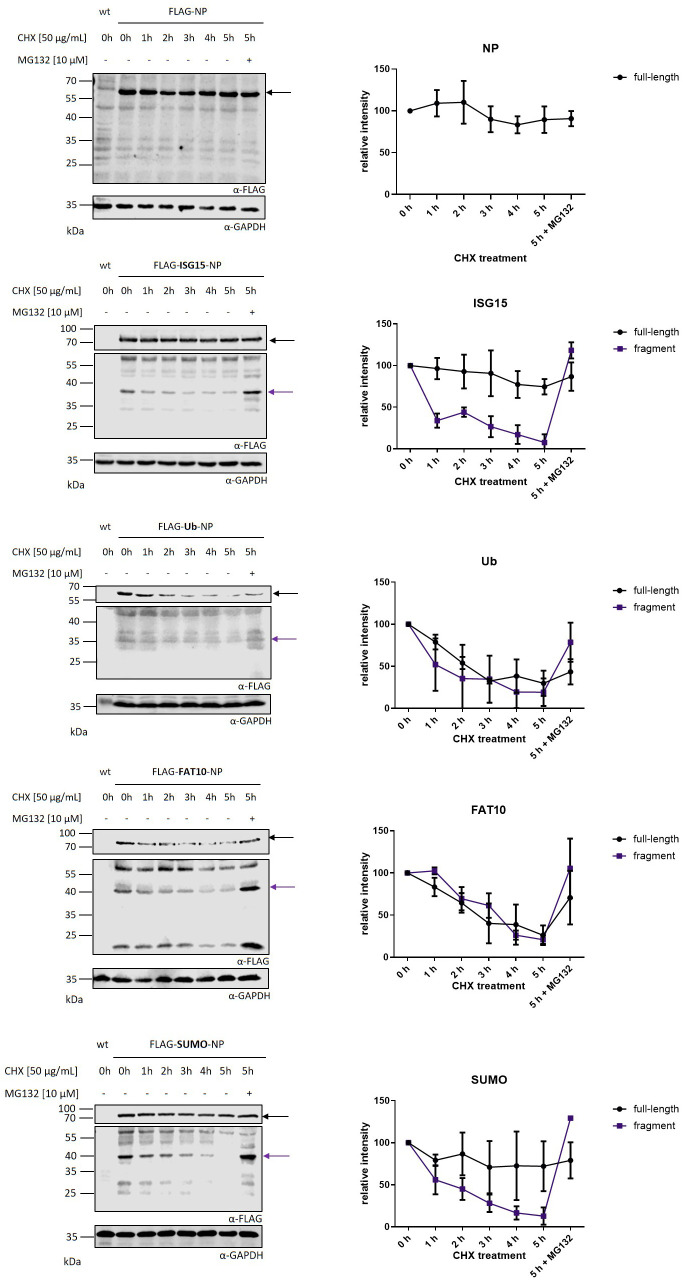
Kinetics of nucleoprotein degradation. Cycloheximide (CHX) chase analysis of full-length protein and protein fragments after transient transfection of HEK293T cells with expression constructs for indicated FLAG tagged nucleoprotein constructs. Before harvesting, cells were treated for indicated time points with 50 µg/ml CHX to inhibit protein *de novo* synthesis. Proteasomal activity was inhibited by adding 10 μM MG132 for 5 h of CHX chase. Shown on the left are representative FLAG western blots of three independent experiments. GAPDH was used as loading control. Black arrow indicates full-length protein and purple arrow indicates DRiP fragment. Graphs on the right side show the quantification of FLAG signals as the mean ± SEM relative to GAPDH derived from three independent experiments (n=3).

To further characterize the observed fragments, the degradation rate of N-terminally modified LCMV-NP constructs was analyzed. HEK293T cells were transiently transfected with plasmids encoding FLAG tagged LCMV-NP constructs and cycloheximide chase experiments were performed (**Figure 3**). Full-length Flag-NP, Flag-ISG15-NP, and Flag-Sumo-NP were barely degraded within 5 hours. In agreement with previous data, FAT10 (16, 26) and ubiquitin (13, 26) accelerated the degradation of the full-length NP. Fusion of ISG15, Sumo, FAT10 and ubiquitin to the NP, led to a degradation of the observed fragment within 5 hours. Similar to **Figure 1**, the degradation was proteasome dependent. Taken together, the dependency of the observed fragments on neosynthesis together with their short half-life, and the degradation by the proteasome implicates these fragments as “Defective Ribosomal Products” or shortly, DRiPs.

### Cell stress leads to an increased DRiP production

**Figure 4:**
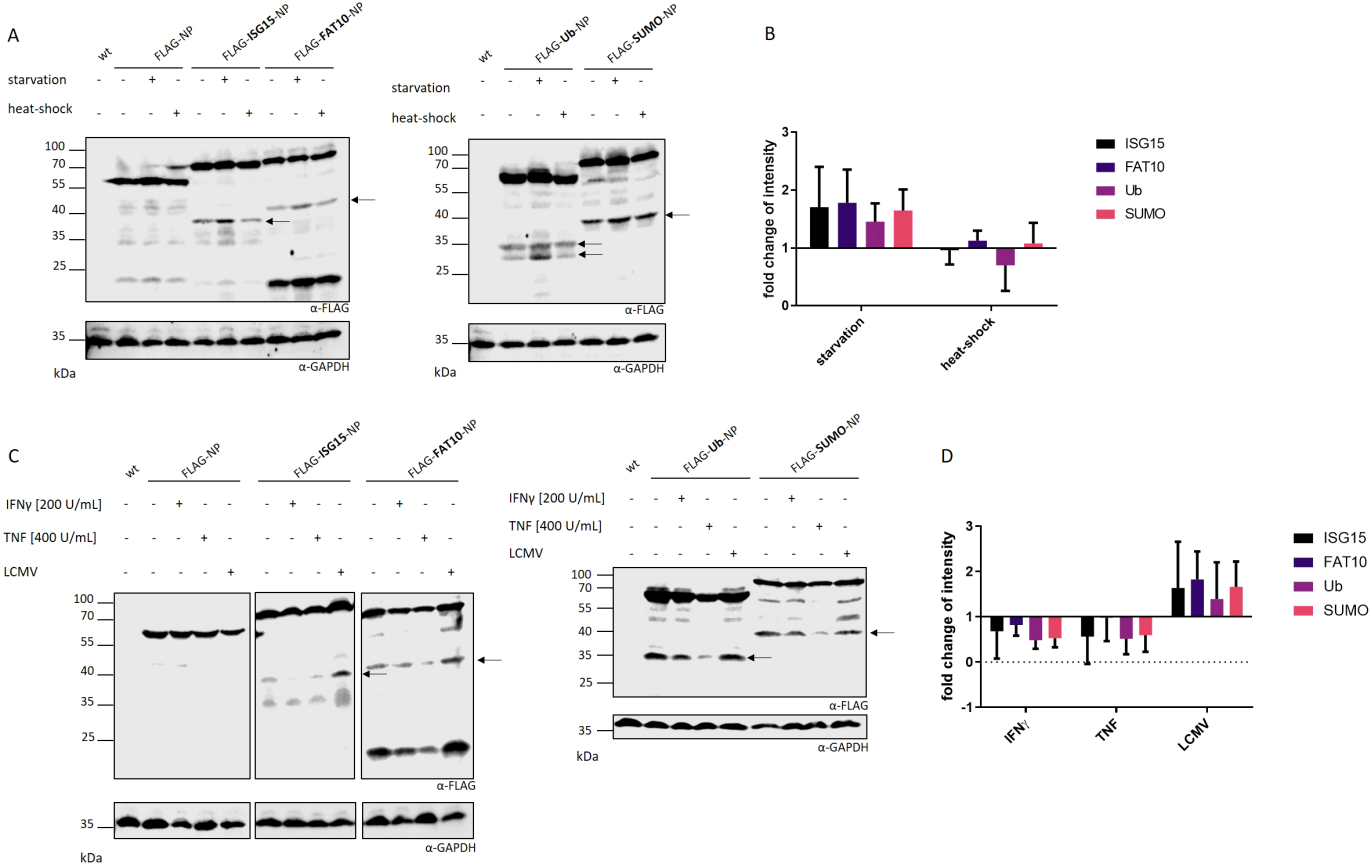
Impact of cellular stress on DRiP formation. The formation of DRiPs was studied under conditions of **(A)** FCS starvation and heat-shock or **(C)** viral infection (LCMV-WE, MOI:10), IFNγ treatment, or TNF treatment in HEK293T cells transiently transfected with indicated constructs. DRiP fragments are visualized by FLAG western blot. Representative western blots of three independent experiments are shown. Proteasomal activity was inhibited with 10 µM MG132 for 5 hours. **(B, D)** Quantification of FLAG signals after **(B)** FCS starvation and heat-shock or **(D)** LCMV infection, IFNγ treatment, or TNF treatment as the mean ± SD derived from three independent experiments (n=3). For quantification of the band intensity, the corresponding full-length FLAG-tagged protein was used for normalization. **(B, D)** Statistical analysis did not reveal significant differences.

Previous studies showed that cell stress has a negative impact on translation fidelity, leading to an increased production of DRiPs in type 1 diabetes (19). An extended translatome in human pancreatic β-cells could be found upon local inflammation leading to increased visibility of β-cells to immune cells (27, 28). IFNγ treatment distorted translation fidelity, triggering the generation of alternative translation initiation sites. Therefore, we wanted to examine whether different cellular stress conditions, including FCS starvation, IFNγ treatment, TNF treatment, viral infection (LCMV) and heat-shock, lead to enhanced DRiP formation in our set-up. HEK293T cells were transiently transfected with plasmids encoding the different FLAG tagged NP constructs and subjected to different stress conditions. The synthesis of DRiPs was monitored by immune blotting. To quantify DRiP fragments, MG132 was used to prevent protein degradation. While heat-shock showed no effect on DRiP formation, cytokine treatment with IFNγ or TNF even had a slight negative impact by reducing the accumulation of the DRiP fragments (**Figures 4C, D**). Interestingly, we observed that FCS starvation and infection with LCMV-WE could enhance the production of DRiP fragments (**Figures 4A, C**). Both stress conditions led to an accumulation of fragments, which was up to two-fold higher compared to untreated controls (**Figures 4B, D**). One possible explanation for the increased production of DRiP fragments could be an altered translation. Since ribosomes are highly regulated protein complexes they are affected by environmental changes including cellular stress (28, 29). It could be shown that IFNγ treatment affects translational fidelity leading to neoantiges presented on MHC I molecules (28). To investigate whether cell stress has an impact on actively transcribing ribosomes, polysome profiling was performed with cells subjected to different cellular stress conditions. In polysomes profiles, no shift from heavy polysomes to monosomes in IFNγ-treated or starved cells could be observed (**Supplementary Figures 2A, B**). This indicates no effect of the tested stress conditions on translational fidelity in polysome profiling assay. For control, cells were treated with puromycin, a protein synthesis inhibitor. Here, the profile showed a shift toward monosomes due to ribosome stalling (**Supplementary Figure 2D**). As expected, profiles from cells, which underwent heat-shock also showed a shift from heavy polysomes to monosomes, indicating translational repression (**Supplementary Figure 2C**). Together, these data show that cell stress in form of FCS starvation or viral infection leads to an increased DRiP production.

### FCS starvation increases MHC class I expression

**Figure 5:**
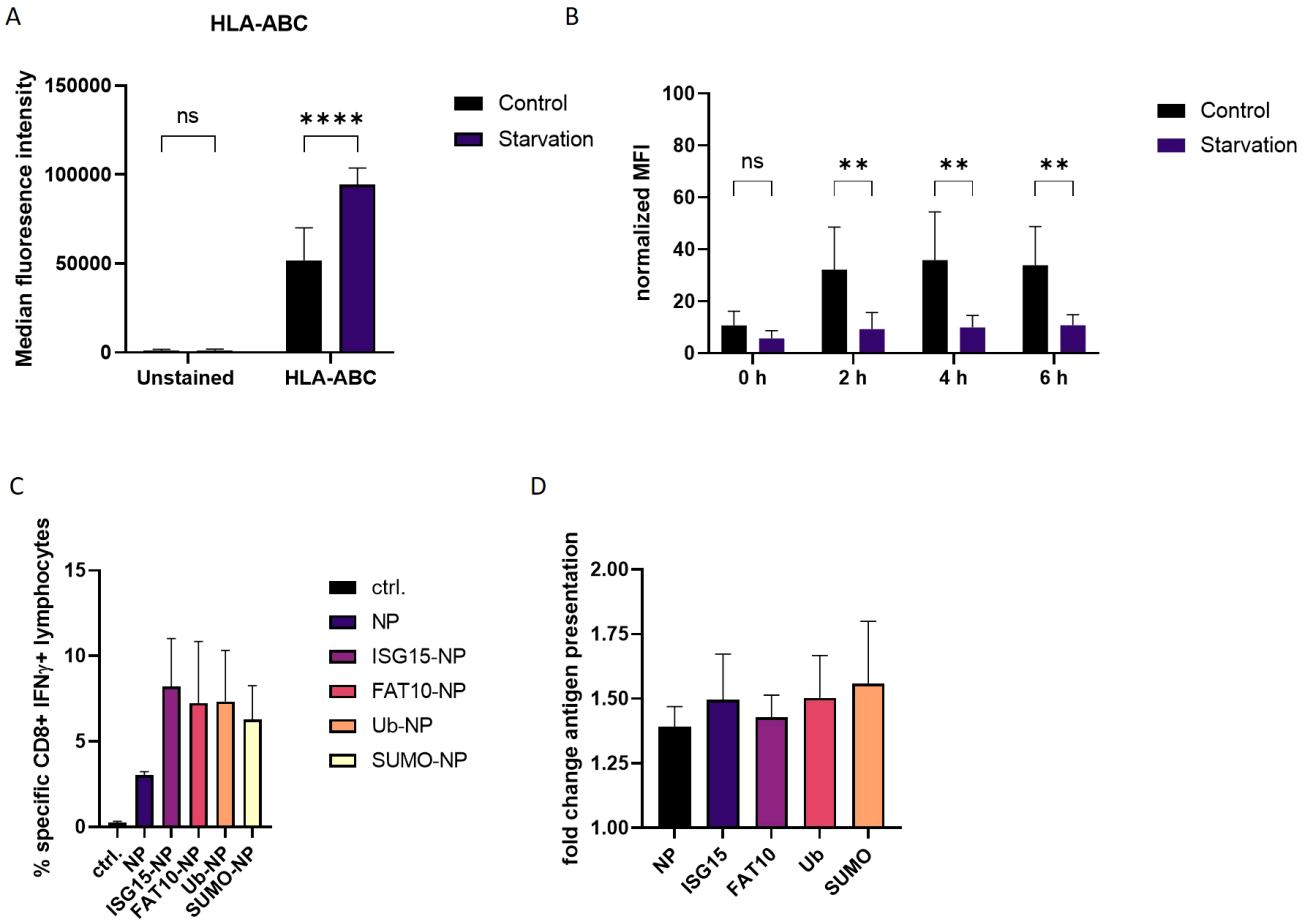
Effect of FCS starvation on MHC-I antigen presentation. **(A)** HLA-ABC surface staining of HEK293T cells, which were exposed to FCS-free culture medium or complete culture medium (control) for 24 h. **(B)** Total human MHC class I surface expression was analyzed via flow cytometry at indicated time points after acid wash treatment. HEK293T cells were subjected to FCS starvation or complete culture medium (control). Untreated cells (no acid wash) served as reference for maximal MHC class I surface expression and were set to 100**%**. **(C)** HEK–Ld cells were transiently transfected with expression constructs for LCMV nucleoprotein or N-terminally modified nucleoprotein. After 24 h, HEK-Ld cells were co-cultured with NP118–126-specific CD8+ T cells for 5 h and analyzed by flow cytometry after staining for CD8 and intracellular IFNγ. Shown are the mean percentages ± SD (n=3) of IFNγ-positive cells of CD8+ cells (y-axis). **(D)** HEK-Ld cells transiently transfected with indicated constructs were used to stimulate NP118–126-specific CD8+ T cells. 5h after transfections, cells were subjected to FCS starvation (indicated stav) or left untreated. 24 hours post transfection indicated numbers of HEK-Ld were exposed to NP118–126-specific CD8+ T cells for stimulation. Activation of NP118–126-specific CD8+ T-cells was analyzed by double staining for surface CD8 and intracellular IFNγ. The percentage of IFNγ-positive cells of CD8+ cells is shown as determined by flow cytometry. Graph shows quantification of CD8-IFNγ double positive cells as detected by flow cytometry. For quantification, untreated cells were used as reference. Data is shown as mean ± SD derived from three independent experiments (n=3). Two-way ANOVA with Sidak**’**s multiple comparisons test. For normalized data, two-way repeated measures ANOVA with Sidak**’**s multiple comparison test was applied for analysis. **p < 0.01; ****p < 0.0001 was applied for statistical analysis.

Ribosomal infidelity increased by cellular stress can result in the generation of neoantigens in human β-cells (29). DRiPs generated during cytokine treatment served as a pool for novel antigens presented by MHC class I molecules resulting in enhanced MHC class I surface expression (19, 28). These studies indicate the involvement of nonconventional translation processes in autoimmune diseases and viral infection. We found that FCS starvation increased DRiP formation in our system (**Figures 4A, B**). To investigate whether cellular stress in form of FSC starvation alters MHC-I antigen presentation, MHC class I surface expression was measured. HEK293T cells were subjected to FCS free medium and an acid wash treatment was performed to remove MHC class I–peptide complexes from the cell surface. The reappearance of the MHC class I molecules was measured by flow cytometry. Interestingly, we already observed a significantly higher MHC class I levels on the surface of FCS starved cells before the acid wash treatment compared to control cells (**Figure 5A**). Additionally, the reappearance of MHC class I molecules was markedly reduced in FCS starved cells compared to controls (**Figure 5B**). This effect was observed for pan HLA-ABC (**Figures 5A, B**) as well as for HLA-A2 (**Supplementary Figure 3**). To exclude increased cell death post FCS starvation, a dead cell staining using propidium iodide (PI) was performed (**Supplementary Figure 4**). Although acid wash slightly increased cell death compared to untreated cells, no significant difference in starved cells compared to control cells 6 hours after acid wash treatment could be observed. Furthermore, analysis of MHC-I surface expression was restricted to living cells according to forward scatter/side scatter in flow cytometry. These data are consistent with a previous study, which did not observe caspase-3 activation in FCS starved HEK293 cells (30). To exclude that FCS starvation in general alters expression of surface molecules, cells were stained for the transferrin receptor CD71 (**Supplementary Figure 4B**). No difference could be observed for CD71 after 24 h FCS starvation. Since cell stress leads to higher DRiP production, which in turn contributes to MHC class I restricted antigen presentation, we further investigated the role of FCS starvation on the generation of MHC class I ligands (12, 31).

Therefore, HEK293T cells stably expressing murine MHC molecules H-2L^d^ (Hek-Ld) were transiently transfected with plasmids encoding the NP alone or N-terminally fused to ubiquitin or different ULMs (**Figure 1B**; **Supplementary Figure 5**). Co-transfection of a plasmid encoding GFP showed that transfection efficiency of the different constructs was similar (**Supplementary Figure 5**). One day post transfection, Hek-Ld cells were co-cultured with splenocytes derived from LCMV infected BALB/c (H-2d) mice in order to stimulate NP118–126-specific cytotoxic T lymphocytes (CTLs). Of note, the LCMV-specific T cell response in BALB/c is pre-dominantly directed to NP118–126 (32). Recognition of NP118–126 presented on Hek-Ld cells leads to the activation of NP118–126 specific T cells which can be measured by double staining for CD8 and intracellular IFNγ and analysis by flow cytometry (**Supplementary Figure 6**). Similar to our previous findings (13, 26, 33), the presentation of NP118–126 was enhanced when ubiquitin or other ULMs were N-terminally conjugated to the NP compared to the expression of the NP alone (**Figure 5C**). To investigate the influence of cell stress on the antigen presentation, we repeated the experiment and subjected the cells to FCS free medium before encounter with splenocytes. Interestingly, presentation of the LCMV nucleoprotein epitope NP118–126 was notably increased when antigen-presenting cells underwent FCS starvation (**Figure 5D**). This effect could be observed for all constructs, thus indicating a general impact on the generation of the LCMV specific epitope NP118–126 (**Figure 5D**), which is consistent with the increased MHC-I surface expression after FCS starvation (**Figures 5A, B**). Taken together, these data show that cellular stress in form of FCS starvation can increase MHC class I surface expression, consistent with an increased presentation of the viral NP118–126 epitope.

## Discussion

The defective ribosomal protein (DRiP) hypothesis was introduced in 1996 when the question arose how MHC-I peptides were generated within one hour from rather stable viral proteins in the context of a viral infection (3, 26). The prematurely terminated polypeptides were proposed to explain the immunogenicity of influenza A virus-derived peptides detected prior to synthesis of their source proteins in virally infected cells. DRiPs were first described as non-functional or imperfectly made proteins, resulting from premature termination during translational but also include proteins that are unable to fold properly although correctly translated (3). Over time, the definition was expanded and now includes also unfunctional proteins resulting from defective mRNAs, frame shifting, tRNA-amino acid misacylation and all other errors that occur during translation (4–6). Therefore, DRiPs make an important source for MHC class I ligands. Indeed, large numbers of HLA class I peptides were observed to be derived from DRiPs (34). The LCMV-NP was the first substrate for which a major prediction of the DRiP hypothesis, namely the requirement for neosynthesis, is shown to hold true (12). However, using a different system and model antigen, mature antigen was the major source of peptides presented, thereby excluding a dominant role of DRiPs (35). In our previous work, we found indications that DRiPs are induced by conjugation of a protein to the ubiquitin-like modifier ISG15 (16). N–terminal fusion of ISG15 to the nucleoprotein of LCMV leads to its degradation in a proteasome-independent manner while the antigen presentation of LCMV-derived epitopes is strongly dependent on the proteasomes, suggesting that the full-length protein is not the source for presentation on MHC-I but the presented peptides rather derive from DRiPs. Here, we confirm and expand our previous findings and show that DRiPs emerge after N-terminal modification of the LCMV nucleoprotein with ISG15 as well as ubiquitin and the other ULMs FAT10 and SUMO (**Figure 1**). The potential DRiPs do not derive from the full-length protein, since C-terminal fragments could not be detected (**Figure 2A**). The fragments rather result from aborted translation. In our previous findings, we could show that the N-terminal conjugation of ISG15 to the nucleoprotein of LCMV could enhance the peptide presentation of NP396–404 (16). Here, we only investigated the epitope NP118–126, which is comprised in the here identified DRiP fragment (**Figures 1A, B**). Since we could not detect any C-terminal fragments it can be assumed that the here identified fragments occur as a result of premature translational termination. A further downstream processing of the full length protein by i.e. proteases is therefore rather unlikely. Noteworthy, the further downstream epitope NP396–404 cannot be produced from the DRiP fragments described here. Therefore, the NP396–404 must be generated from DRiPs, which cannot be detected in our western blot experiments. The DRiP fragments derived from the different modifiers have a similar half-life, which is notably shorter than their stable native full-length protein. Fragments are strongly stabilized by proteasome inhibition (**Figure 3**). Interestingly, the observed DRiP fragments possess a defined molecular weight distinctive for each construct according to the modifiers molecular size, indicating a specific location on mRNA responsible for translational termination (**Table 1**). To date, it is not clear whether DRiPs solely arise randomly as a consequence of translational errors, or whether they can be actively controlled. A hypothesis posits the existence of immunoribosomes, which generate peptides specifically targeted to proteasomes thereby increasing the efficiency of antigen processing (40). These immunoribosomes are proposed to preferentially translate newly synthesized mRNAs, which are distinguished by a unique pattern contributing to the detection of viruses (40). However, experimental evidence for the existence of such immunoribosomes is scarce. Since different N–terminal modification led to fragments with a putatively similar C–terminus, a random DRiP generating mechanism seems unlikely. It seems that the modified N–terminus in the context of the LCMV-NP induces a structure that leads to a pre-mature translation termination. Whether secondary structures on the mRNA, the elongating protein itself, or a different mechanism leads to pre-mature translation termination remains to be investigated. Identification of a potential motive resulting in DRiP formation opens up new possibilities for the development of vaccination strategies. In this study, we could show that N-terminal conjugation of ubiquitin and ULMs to the long-lived NP of LCMV leads to its degradation and enhanced MHC class I presentation of the LCMV-derived epitope NP118–126. Thus, modulating proteins used for vaccination with a motive inducing DRiPs could further enhance the CTL response to the vaccinated protein.

**Table 1:** Table showing the molecular weight (MW) of the respective DRiP fragment and its modification.

Construct	Estimated MW of DRiP fragment (kDa)	MW modifier (kDa)	MW 6xHis3xFLAG tag (kDa)	MW fragment – size modification (kDa)
FLAG-ISG15-NP	40	15 (36)	3,7	21,3
FLAG-Ub-NP	36	8,6 (37)	3,7	23,7
FLAG-FAT10-NP	43	18 (38)	3,7	21,3
FLAG-SUMO-NP	40	12 (39)	3,7	24,3

The size of DRiP fragments were estimated from the FLAG western blot in **Figure 1**. Molecular weight of modifiers were taken from literature as indicated.

Earlier studies regarding type 1 diabetes pathogenesis could demonstrate an impact of cell stress on protein stability in the context of inflammation (19, 41). The inflammatory milieu in human pancreatic islets caused translational errors generating highly immunogenic insulin gene–derived polypeptides, which can be recognized by autoreactive T cells leading to the destruction of β-cells (19, 28, 41). Specific killing was observed during inflammation, although no difference in the amounts of DRiPs could be detected in cytokine-treated and untreated samples (29). Differently, another study showed that interferon treatment increased DRiP peptides in the HLA peptidome by about two-fold (42). Interestingly, such typical DRiP–derived MHC peptides were from the surplus subunits of the proteasome and ribosome, which are degraded because of the transition to immunoproteasomes and a new composition of ribosomes incorporating protein subunits that are induced by the interferon. Our data strongly suggest an impact of cell stress on DRiP synthesis. In contrast to cytokine treatment, FCS starvation as well as LCMV infection resulted in an up to two-fold increased DRiP formation (**Figure 4**). A possible reason for the impaired translational fidelity could be a reduced priority for protein translation in the stressed cell in order to focus on catabolic processes to promote cell survival. Serum starvation reduces basal cellular activity (43). However, the impact is unpredictable and depends on cell types. In the course of a viral infection, virus-infected cells are hijacked by the virus to massively produce virus progenitors. Generation of viruses and activation of immune cells leads to an enhanced production of newly synthesized proteins and, thus, to increased protein degradation to maintain protein homeostasis (44). Hence, these processes can promote translational infidelity thereby promoting DRiPs.

An FCS depleted environment leads to a remarkable increase in the production of small peptide fragments (**Figures 4A, B**). It is further known that DRiPs contribute to the MHC class I peptide pool. Indeed, a recent study could show that inflammation induced cell stress leads to an increased activation of DRiP specific CD8 cells (19). This finding underlines the importance of enhanced expression of MHC molecules in activating cytotoxic T cells resulting in destruction processes. In our experimental settings, we observed an effect of cell stress on the display of MHC class I molecules on the cell surface. There, a significant increase in MHC class I surface expression was detected in cells subjected to FCS starvation (**Figure 5A**). Of note, the recovery of MHC molecules was significantly slower for stressed cells compared to untreated cells after the acid wash (**Figure 5B**). This indicates that starvation increases the antigen presentation on MHC class I molecules after 24 h, but is rather detrimental for short-term antigen presentation. Cell stress in form of starvation can increase autophagy. However, in a previous study inhibiting autophagy had no impact on the presentation of SIINFEKL peptides from a NEDD8-Ovalbumin fusion protein (18). Nutrient or serum deprivation inhibits mammalian target of rapamycin (mTOR) and stimulates protein breakdown by inducing autophagy, which provides the starved cells with amino acids for protein synthesis and energy production (45). An increased protein degradation provides a larger peptide pool for antigen presentation that could lead to an enhanced MHC class I surface expression (**Figure 5A**). Furthermore, inactivation of mTOR increases overall protein degradation by the ubiquitin proteasome system by enhancing the ubiquitination of many cell proteins (46). It remains to be investigated whether the mTOR pathway is involved in up-regulation in MHC-I surface expression in our system. In case of the recovery of peptide-MHC class I complex on a shorter timescale, starvation seems to be rather hindering. Polysome profiles showed no visible differences after exposure of cells to IFNγ treatment or starvation indicating unaltered ribosomal activity (**Supplementary Figures 2A, B**). General translational repression could be seen in puromycin-treated cells, which is known to inhibit protein synthesis by inducing premature termination. In polysome profiles of heat-shocked cells a shift toward monosomes was observed which may occur due to impaired translational fidelity during heat shock (**Supplementary Figure 2D**). Western Blot analysis of DRiP fragments under different stress conditions showed no increase upon heat shock treatment (**Figure 4**). This suggests that the translational fidelity does not influence the occurrence of DRiP fragments. Furthermore, if only a small fraction of ribosomes undergoes translational repression triggered by cellular stress, this method may be not suitable to detect minor differences in translational processes. Similar fragment sizes indicate termination of translation at the same position within the LCMV nucleoprotein (**Table 1**). Ribosomes might disassembles due to secondary structure formation of mRNA or a motive within the nucleoprotein RNA sequence, which is not present in the “non-fused” NP. This premature termination cannot be monitored by polysome profiling since it is not affected by cell stress induced ribosome stalling. We previously reported that the presentation of the LCMV nucleoprotein derived epitope NP118–126 is increased when the nucleoprotein is N-terminally modified with ISG15, ubiquitin or FAT10 (16, 26), which could be confirmed in this study (**Figure 5C**). In addition, we found an increase in the presentation of the nucleoprotein specific epitope NP118–126 when antigen–presenting cells were subjected to FCS starvation (**Figure 5D**). Previous studies described a higher T-cell specific killing under inflammatory conditions as well as enhanced DRiP production as a result of an alternative translatome (19). Similarly, exposure of cells to stress in form of FCS depletion led to an increased DRiP formation and antigen presentation. Since the small fragments synthesized during cell stress are proven to be unstable and degraded by the proteasome, they may represent important by-products of the translation process that can contribute to peptide generation for presentation on MHC class I molecules. The fact that translation is a highly regulated process and affected by environmental cues suggests that disturbances occurring in cells may alter the cellular translation machinery, preferentially synthesizing DRiPs in order to evoke immune surveillance.

Taken together, our findings illustrate the importance of protein stability and degradation on MHC class I antigen presentation during cellular stress.

## Materials and methods

### Mice, cell lines and media

BALB/c (H-2d) mice were originally obtained from Charles River Laboratories Germany and further bred in the animal facilities of the University of Konstanz. Mice were kept in a specific pathogen-free facility and animal experiments were approved by the Review Board of Governmental Presidium Freiburg of the State of Baden-Württemberg, Germany (I-21/001). 8–10 week old mice were used for all experiments. For LCMV infection, 200 pfu LCMV-WE were injected intravenously (i.v.).

The human embryonic kidney cell line HEK293T (ATCC, USA), Hek-Ld (HEK cells stably transfected with a plasmid encoding H-2Ld, kindly provided by A. Bitzer) were cultured in DMEM supplemented with 10% FCS, 100 U/mL penicillin, and 100 μg/mL streptomycin. For starvation experiments, FCS was omitted from the media. When heat-shock experiments were performed, cells were incubated at 42°C for 30 minutes. For cytokine stimulation, cells were treated with IFNγ (200 U/mL) or TNF (400 U/mL) for 24 hours. For infection, cells were incubated with LCMV-WE virus (MOI = 10) for 24 hours. Culture media and supplements were obtained from Thermo Fisher Scientific (Germany). Splenocytes and isolated primary murine CD8+ T cells were maintained in IMDM containing 10% (v/v) FCS, 100 U/mL penicillin and 100 μg/mL streptomycin and 50 μM β–mercaptoethanol.

### Generation of LCMV nucleoprotein constructs

Plasmids encoding the different His3xFLAG-tagged LCMV nucleoprotein fusion proteins were cloned by polymerase chain reaction (PCR). The His3xFLAG was amplified by PCR using the primer pair: 5’-GTACTACTCGAGATGGGCCATCATCATCATC-3’ (forward) and 5’-TTAATTCTCGAGCTTGTCATCGTCATCCTTGTAATC-3’ (reverse). The fragment was digested with XhoI and inserted into the XhoI site of the pCMV vector encoding N-terminally modified LCMV nucleoprotein (16). For the introduction of the C-terminal HA tag, the Q5^®^ Site-Directed Mutagenesis Kit was used according to the manufacturer’s instructions (New England Biolabs). Shortly, the plasmids encoding the LCMV expression constructs were amplified by PCR with the following primers containing the sequence of the HA tag: 5’-GCCCGACTACGCCTAAGCGGCCGCGGGGATC-3’ (forward) and ACGTCGTAGGGGTAGAGTGTCACAACATTTGGGCCTCTAAAAATTAGG (reverse). After PCR, the amplified material underwent a Kinase-Ligase-DpnI (KLD) reaction for circularization prior to transformation.

### Transfection of cell lines

Transfection of HEK293T and Hek-Ld cells was performed using polyethylenimine (PEI, Polysciences) with a 1:5 (µg/µg) ratio of DNA to reagent.

### Cycloheximide chase experiment

HEK293T cells were transiently transfected with plasmids encoding the His3xFLAG-tagged LCMV-NP (FLAG-NP) expression constructs. 24 hours post transfection, cells were treated with 50 μg/mL cycloheximide (CHX) for 5 hours. For control, one sample was additionally treated with 10 µM MG132. Cells were collected every hour and lysed in RIPA lysis buffer (50 mM Tris, pH 7.5, 1 mM EDTA, 150 mM NaCl, 0.1% SDS, 1% NP-40, 1x protease inhibitor cocktail (complete Mini EDTA-free; Roche, Mannheim, Germany). After incubation for 30 minutes on ice, lysates were cleared by centrifugation at 14000 x g for 20 minutes at 4°C. Samples were stored at 20°C for later analysis or directly used for SDS-PAGE and western blotting.

### SDS-PAGE and western blotting

SDS-PAGE and western blotting was performed as described previously (47, 48). Shortly, proteins of cleared lysates were denatured by incubating with SDS sample buffer (50 mM Tris-HCl pH 6.8, 2% SDS, 10% glycerol, 20 mM dithiothreitol, 0.02% bromophenol blue) for 5 minutes at 95°C. Proteins were separated by 12% Tris-Glycine polyacrylamide gels and transferred onto nitrocellulose membranes (0.45 µm, GE HealthCare). Membranes were blocked for 1 hour using Intercept Blocking Buffer (LI-COR) prior to incubation with primary antibodies at 4°C over night. Following primary antibodies were used: anti-Flag (clone F7425, Sigma), anti–HA (clone HA–7, Sigma) and anti–GAPDH (clone G9545, Sigma). Next, membranes were washed with TBS-T (20 mM Trizma^®^base, 137 mM sodium chloride and 0.015% Tween^®^20) and incubated with appropriate secondary antibodies. Secondary antibodies: IRDye 680RD goat anti–mouse IgG and IRDye 800CW goat anti-rabbit IgG (both LI-COR). Blots were developed using Odyssey Fc Imaging System (LI-COR) and quantified using ImageStudio (Ver. 5.2, LI-COR).

### Acid wash and MHC class I surface expression

HEK293T cells were incubated in FCS depleted culture medium for starvation or maintained in complete culture medium for control. After 24 hours, cells were acid washed using citric acid buffer (0.131 M citric acid, 0.066 M NaH_2_PO_4_, pH 3) or left untreated as previously described (49). Cells were washed twice with PBS and medium and were further incubated in medium at 37°C over a time course of 6 hours. Samples were taken every 2 hours and MHC class I surface expression was determined by flow cytometry. Cells were stained with anti-HLA-ABC (W6/32, eBioscience) or anti-HLA–A2 (BB7.2, eBioscience) antibodies. Cells were measured on a FACSVerse flow cytometer (BD Biosciences) and analyzed using FlowJo software (BD Biosciences).

### NP118–126 specific CTLs

Splenocytes derived from LCMV-infected (200 pfu LCMV-WE, i.v.) BALB/c (H-2^d^) mice, which mainly contain NP118–126-specific CTLs, were used on day 8 post infection *ex vivo* to determine presentation of NP118–126 on H-2L^d^.

### *In vitro* antigen presentation assays and intracellular cytokine staining (ICS)

Hek-Ld cells were transiently transfected with plasmids encoding N-terminally modified LCMV nucleoprotein or the nucleoprotein alone. Additionally, a GFP expression construct (#CD511B-1, System Biosciences) was co-transfected in a molar ratio of 4:1 (GFP-plasmid:plasmid of interest) as a control to monitor transfection efficiency. After 24 hours, cells were harvested and the amount of GFP positive cells was measured using flow cytometry. For determination of NP118–126 presentation derived from the LCMV nucleoprotein and the fusion proteins, transfected cells were seeded in a 96-well plate with decreasing densities (4.0 x 10^5^, 1. 3 x 10^5^, and 0.4 x 10^5^ cells/well) in duplicates. 5.0 x 10^5^ splenocytes in the presence of brefeldin A (10 μg/mL) were used to detect NP118–126 presentation. Samples were incubated for 5 hours at 37°C and 5% CO_2._ Intracellular cytokine staining was performed as previously described (50). Shortly, cells were fixed with 4% paraformaldehyde and permeabilized with 0.1% (w/v) saponin. Cells were stained using the following antibodies: anti-CD8a (53–6.7, BD Biosciences), anti–IFNγ (XMG1.2, BD Biosciences). The samples were measured on FASCVerse (BD Biosciences) and flow cytometry data was analyzed with FlowJo software (BD Biosciences).

### Polysome profiling

For polysome profiling, 4 x 10^7^ Hek-T cells were seeded in two 15 cm dishes (2 x 10^7^ per dish) and incubated at 37°C and 5% CO_2_ until reaching approximately 80% confluency. The next day, cells were treated depending on the condition. For FCS starvation, cells were exposed to culture medium without FCS, while for type I interferon response, cells were treated with 1000 U/mL IFNγ. To induce translational instability, cells were treated with 1 µg/mL puromycin. Cells were then incubated for 24 hours at 37°C and 5% CO_2_. In case of heat-shock, cells were incubated for 1 hour at 42°C, followed by a recovery phase of 4 hours at 37°C before cycloheximide treatment. For control, cells were left untreated. To create polysomes that reflect the translational status of the cell, ribosome movement on the mRNA must be reduced to prevent ribosome disengagement. Therefore, cells were pretreated with 100 µg/mL cycloheximide for 5 minutes. Before lysis, cells were collected by centrifugation at 300 x g for 5 minutes at 4°C and washed twice with ice-cold PBS containing 100 µg/mL cycloheximide. After discarding the supernatant, cells were lysed in 500 µL hypotonic buffer (5 mM Tris-HCl (pH 7.5), 2.5 mM MgCl2, 1.5 mM KCl, complete EDTA-free protease inhibitor cocktail (Roche), 100 µg/mL cycloheximide, 2 mM DTT, 200 U/mL RNasin (Promega), 0.5% v/w Triton X-100, 0.5% v/w sodium deoxycholate). After centrifugation at 14 000 x g for 5 min at 4°C, cleared lysates were transferred into new pre-chilled tubes, and the total RNA concentration of the supernatant was measured by NanoDrop at 260 nm. RNA was separated by sucrose density gradient centrifugation. Therefore, the equivalent of 20 x OD 260 nm was loaded onto 12 mL of 10 – 50% sucrose gradient followed by ultra-centrifugation at 230500 x g for 2.5 hours in an SW41 rotor (Beckman Coulter) at 4°C. To avoid any disturbance, the acceleration and deceleration were set to their minimum value. For generating a polysome profile, the OD 260 was continuously recorded with a Piston Gradient Fractionator (Biocomp Instruments) and TriaxTM Flow Cell (Biocomp Instruments).

### Statistical analysis

For statistical analyses, groups from three independent experiments were pooled and analyzed for significant differences as indicated in the graph. Statistical significance was determined using GraphPad Prism software (version 9.5.1. GraphPad, San Diego, CA). Error bars represent mean ± SD unless otherwise stated.

## Data Availability

The raw data supporting the conclusions of this article will be made available by the authors, without undue reservation.
