# Supplementary material for: Cellular stress increases DRIP production and MHC Class I antigen presentation
Source: Front Immunol. 2024 Aug 23;15:1445338. doi: 10.3389/fimmu.2024.1445338 (PMC11377247; doi:10.3389/fimmu.2024.1445338)
Supplement: Supplementary file 1 [file DataSheet1.pdf]

Supplementary information

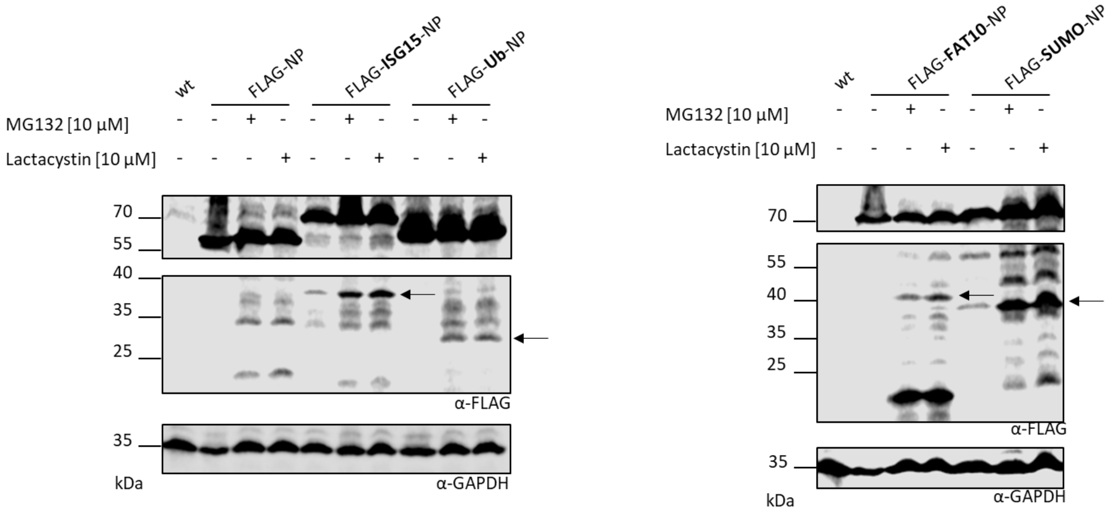

Supplementary Figure 1 Comparison of MG132 and lactacystin on NP fragments. Representative FLAG western blots showing NP fragments after transient transfection of HEK293T cells with indicated expression constructs. Where indicated (+), 24 h post transfection proteasome activity was inhibited by adding MG132 or lactacystin at a concentration of 10 µM for 5 hours. Untransfected cells (indicated wt) were used as negative control. Arrows indicate DRiP fragments. GAPDH was used as loading control (lower western blot).

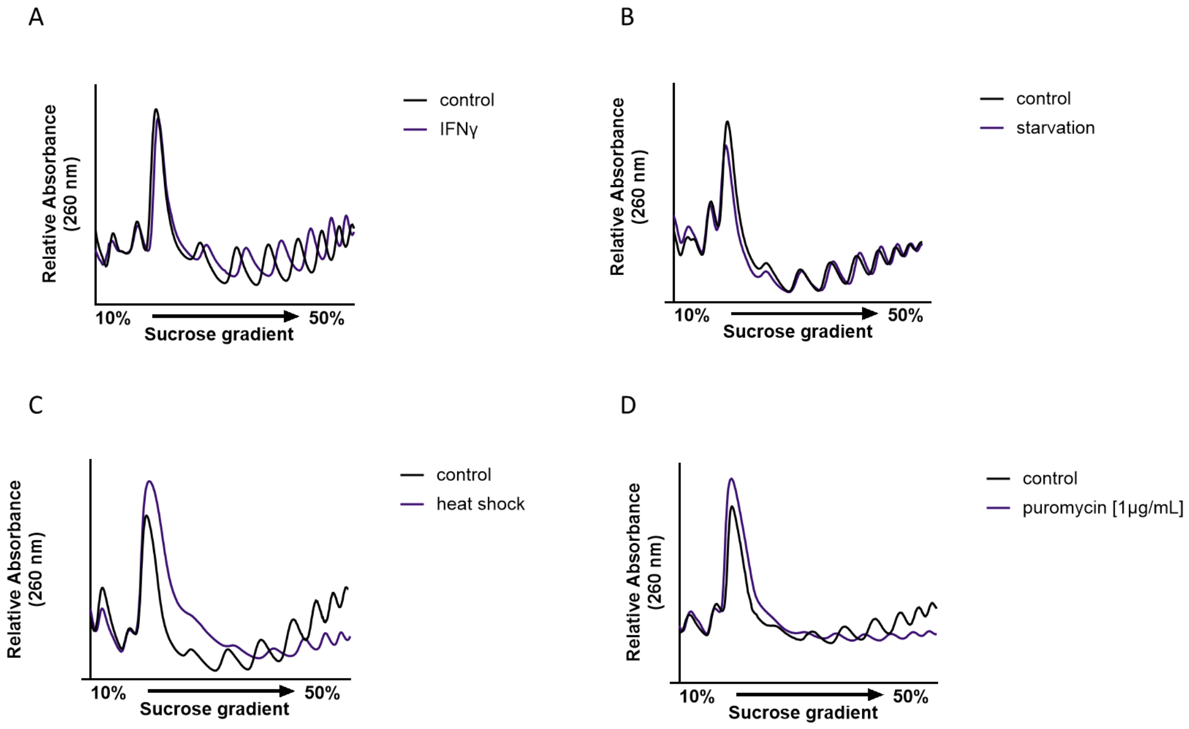

Supplementary Figure 2 Impact of stress conditions on ribosomal activity. Polysome profiles of HEK293T cell subjected to (A) IFNγ, (B) FCS starvation, (C) heat shock, (D) puromycin treatment.

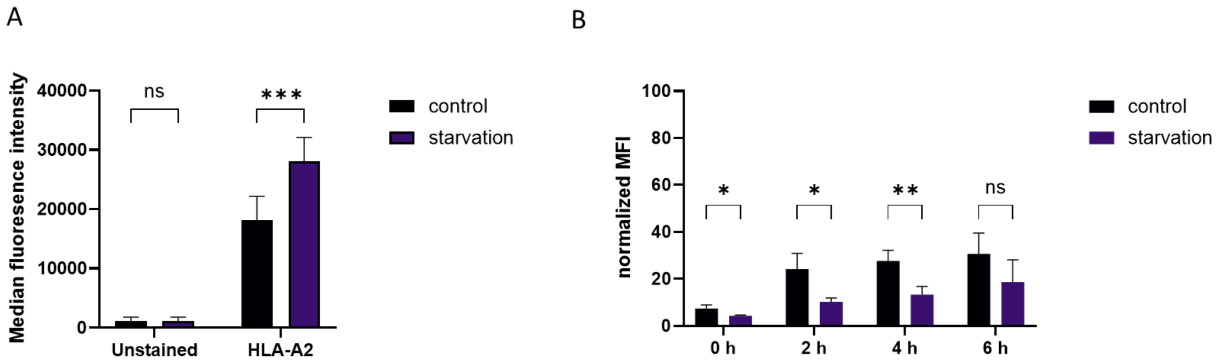

Supplementary Figure 3 Effect of FCS starvation on HLA-A2 surface expression. HEK293T cells were cultured in the absence of FCS to induce cellular stress or were left untreated (control) for 24 hours. (A) HLA-A2 surface expression was determined by flow cytometry. The mean median fluorescence intensity ± SD derived from three independent experiments (n=3) is shown. Two-way ANOVA with Sidak's multiple comparison test, ***p < 0.005 ; ns; not significant. (B) An acid wash treatment was performed to remove MHC-I surface molecules. The recovery of HLA-A2 surface molecules was analyzed via flow cytometry at indicated time points after acid wash treatment. Untreated cells (no acid wash) served as reference for maximal MHC class I surface expression and their median fluorescence intensity (MFI) was set to 100 %. Data is shown as mean ± SD derived from three independent experiments (n=3). Two-way ANOVA with Sidak's multiple comparison test. For normalized data, two-way repeated measures ANOVA with Sidak's multiple comparison test was applied, *p < 0.05; **p < 0.01; ***p < 0.005; ns; not significant.

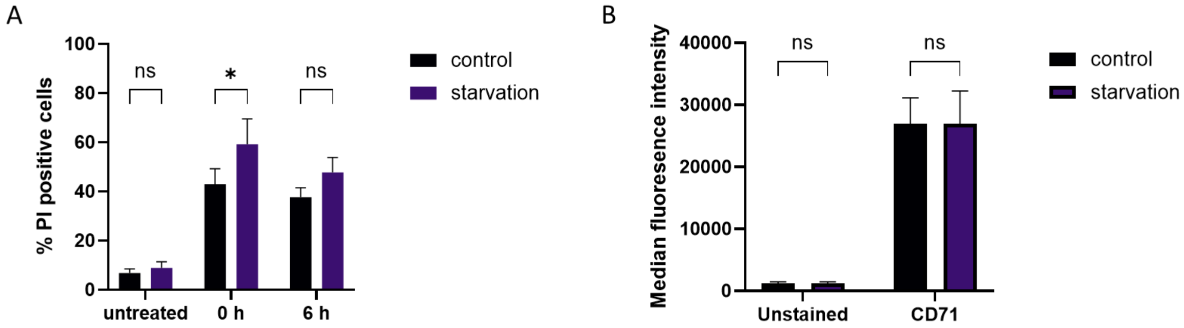

Supplementary Figure 4 Determination of cell viability and expression of the transferrin receptor CD71. HEK293T cells were cultured in the absence of FCS (starvation) or maintained in complete culture medium (control). (A) After 24 hours, an acid wash treatment was performed or cells were left untreated. Cell death was determined by flow cytometry using PI at indicated time points after the acid wash treatment. (B) Expression of the transferrin receptor CD71 was analyzed via flow cytometry. Data is shown as mean ± SD derived from three independent experiments (n=3). Two-way ANOVA with Sidak's multiple comparison test was applied for statistical analysis., . **p < 0.01; ns; not significant.

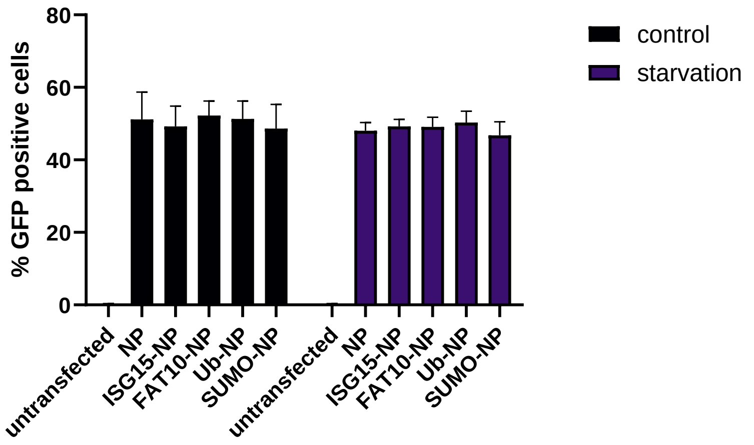

Supplementary Figure 5 Transfection efficiency. HEK-Ld cells were transiently transfected with plasmids encoding indicated constructs. Co-transfection with a GFP expression plasmid served as control for transfection efficiency. The percentage of GFP positive cells was determined by flow cytometry. Data is shown as mean ± SD derived from three independent experiments (n=3).

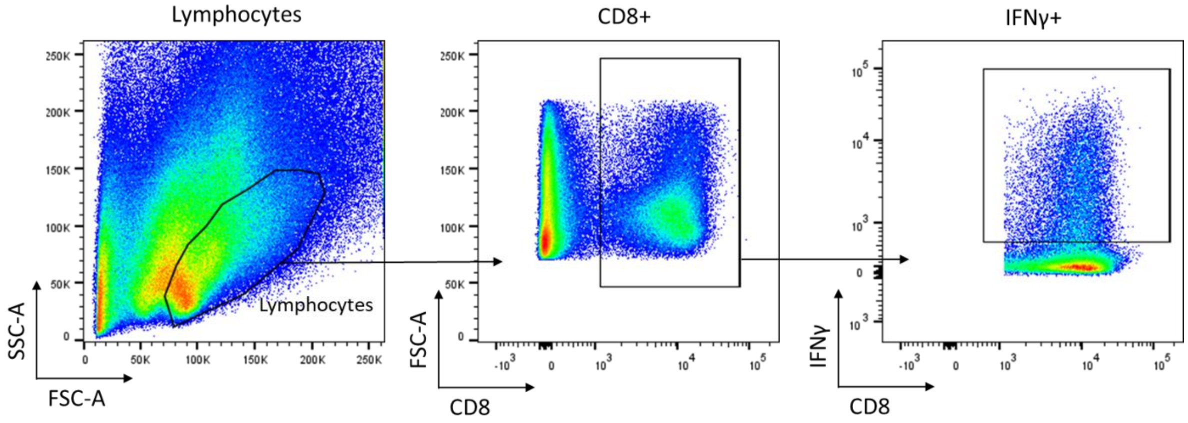

Supplementary Figure 6 Representative flow cytometry dot blots of stimulated splenocytes from LCMV infected BALB/c. Stimulation was achieved by co-culturing with Hek-Ld cells in the presence of the LCMV-derived epitope NP118-126. Activation of T cells was analyzed by staining for surface CD8 and intracellular IFNγ. Percentages of the subset of IFNγ positive cells from the total amount of CD8 positive lymphocytes were determined by flow cytometry.
